# Berberine Facilitates Extinction and Prevents the Return of Fear

**DOI:** 10.3389/fphar.2021.748995

**Published:** 2022-02-03

**Authors:** Shihao Huang, Yu Zhou, Feilong Wu, Cuijie Shi, He Yan, Liangpei Chen, Chang Yang, Yixiao Luo

**Affiliations:** ^1^ Key Laboratory of Molecular Epidemiology of Hunan Province, School of Medicine, Hunan Normal University, Changsha, China; ^2^ Yiyang Medical College, Yiyang, China; ^3^ Department of Forensic Science, School of Basic Medical Science, Central South University, Changsha, China; ^4^ China Hunan Province People’s Hospital, The First-affiliated Hospital of Hunan Normal University, Changsh, China

**Keywords:** berberine, extinction, fear memory, contextual fear conditioning, PTSD

## Abstract

Exposure to a catastrophic event or intense stimulation can trigger fear memories, and the threatening memories persist even over a lifetime. Exposure therapy is based on extinction learning and is widely used to treat fear-related disorders, but its effect on remote fear memory is modest. Berberine, an isoquinoline alkaloid derived from *Coptis chinensis* or *Berberis* spp., has been recently reported to exert a diversity of pharmacological effects on the central nervous system, such as facilitating extinction of drug memory. Here, we explored the effect of berberine on extinction of fear memory using a classical contextual fear conditioning (CFC) paradigm, which is Pavlovian conditioning, can rapidly create fear memories related to contexts. Twenty-four hours or 30 days after CFC training, mice were subjected to context extinction (10 days) to extinguish their behaviors and treated with 12.5 or 25 mg/kg berberine intragastrically 1 or 6 h after each extinction session, followed by reinstatement and spontaneous recovery tests. The results showed that intragastric administration of 25 mg/kg berberine 1 h after extinction significantly promoted the extinction of recent and remote fear memories and prevented reinstatement and spontaneous recovery of extinguished fear in mice. These findings indicate that berberine combined with extinction training could serve as a promising novel avenue for the treatment of fear-related disorders.

## 1 Introduction

Fear response is an emotional experience necessary to adapt to the new environment. However, an exceedingly strong, persistent, and uncontrollable state of fear can lead to the development of anxiety and fear-related disorders, such as posttraumatic stress disorder (PTSD). PTSD is a mental disorder that has a high incidence and difficult to be completely cured, with a lifetime prevalence of 6.8% (Markowitz et al., 2015). The patients exert repeated and long-term fear responses, and this disorder brings great pain to people’s lives and causes a huge social–economic burden.

In the laboratory, fear learning is usually studied by classical (Pavlovian) association between a neutral conditioned stimulus (CS) and a fearsome stimulus (usually one or more footshocks; unconditioned stimulus [US]). After being conditioned to a US, a CS can elicit a fear response (Gutberlet et al., 1902; Mcgaugh, 2000). However, extinction of conditioned fear occurred when repeatedly exposed to a CS without US presentation, which has been proposed to be a new CS–no US memory termed as “extinction memory” (Pavlov, 1927; Morgan and Ledoux, 1999; Berman and Dudai, 2001; Maren et al., 2013; Singewald and Holmes, 2019). Extinction is not the erasure of the original fear memory, but the formation of a new extinction memory to inhibit the expression of fear (Orsini and Maren, 2012; Izquierdo et al., 2016; Likhtik and Johansen, 2019; Bouton et al., 2021). The new extinction memory also involved acquisition, consolidation, and storage processes (Millan et al., 2011; Orsini and Maren, 2012; Maren et al., 2013; Careaga et al., 2016; Knox, 2016). Studies demonstrate that PTSD subjects lead to extinguished fear relapses due to impaired recall of extinction memory (Vervliet et al., 2013; Goode and Maren, 2014; Qin et al., 2021). Therefore, enhancing consolidation of extinction memory has been considered as a strategy for attenuating the original fear memory. Nevertheless, it is easy to relapse because of the persistence of fear memories in humans and rodents, the response of previously extinguished fear will reemerge when the original US is given unexpectedly, called “reinstatement” (Haaker et al., 2014; Goode and Maren, 2019). Similarly, such responses will reoccur when a substantial amount of time has passed, called “spontaneous recovery” (Rescorla, 2004; Lacagnina et al., 2019). Fear memory was subdivided into recent and remote fear memory after training 1 or 30 days in animal studies, respectively (Anagnostaras et al., 1999; Pan et al., 2020; Vetere et al., 2021). Exposure therapy based on extinction theory described above has less effect on remote fear memory and seldom suppresses the reinstatement and spontaneous recovery of extinguished fear (Costanzi et al., 2011; Graff et al., 2014; An et al., 2017). Also, Dudai’s remarkable discovery demonstrated that memories become impervious to interferences that disrupt synaptic consolidation within 6 h after the training, which provides a precise time window to interfere with consolidation memory (Dudai, 2004). Together, there is an urgent need to enhance the efficacy of exposure therapy and develop potential and effective strategies for fear-related disorders treatment.

Berberine is a biologically active isoquinoline alkaloid extracted from the roots or stems of the Chinese traditional medicine *Coptis chinensis* and *Berberis* spp. (Neag et al., 2018; Belwal et al., 2020). It was used as a traditional medicine for centuries and has been clinically widely used as an over-the-counter (OTC) drug for gastrointestinal infections (Domitrovic et al., 2011; Habtemariam, 2020). Recently, growing evidence demonstrates that berberine has therapeutic potential in many central nervous system pathological conditions. Berberine exerted antidepressant-like effect by modulating brain biogenic amines including norepinephrine, serotonin, and dopamine (DA) (Kulkarni and Dhir, 2008; Habtemariam, 2020) and showed neuroprotective effect via modulation of Sirt 1/p38 MAPK expression in traumatic brain injury (Wang et al., 2018). In addition, berberine improves brain DOPA/DA levels to ameliorate Parkinson disease by regulating gut microbiota (Wang et al., 2021). Moreover, berberine modulates oxytocin receptors to attenuate methamphetamine consumption and anxiety-like behaviors (Alavijeh et al., 2019). Furthermore, berberine has been reported to alleviate anxiety-like behavior in rats with posttraumatic stress disorder and facilitate extinction of drug memories (Lee et al., 2018; Shen et al., 2020). In Shen and colleagues’ study, they found that brain-derived neurotropic factor (BDNF) and AMPA receptors (GluA1 and GluA2) were associated with enhanced extinction memory consolidation (Shen et al., 2020). AMPA receptor GluR1 signaling is also involved in consolidation of remote fear memories (Andero and Ressler, 2012). Although fear memory and drug memory are different types of memory, they have similar memory processes, underlying neurobiological mechanisms, and both are driven by emotional or traumatic experiences, and thus both of them are considered as pathological emotional memories (Torregrossa et al., 2011; Taylor and Torregrossa, 2015). Besides that, much evidence shows the levels of BDNF, GluA1, and GluA2 deemed for critical roles in both drug extinction memory and fear extinction memory (Yu et al., 2009; Andero and Ressler, 2012; Rakofsky et al., 2012). For the reasons mentioned previously, it is reasonable to speculate that berberine may have effect on fear extinction memory. However, the effects of berberine treatment on the extinction of fear memory and its therapeutic potential for fear-related disorders have not been reported yet.

Taken together, the data to date suggest a hypothesis that berberine plays a critical role in fear extinction memory. Here, we first aimed to evaluate the optimum dose of berberine and then tested the hypothesis that berberine combined with extinction training enhances extinction memory and prevents the return of fear. In the present study, we used a classical contextual fear-conditioning (CFC) model to test the effect of berberine treatment on extinction of both recent and remote fear memory and investigated whether berberine combined with extinction training could persistently attenuate and prevent the return of fear in mice.

## 2 Materials and Methods

### 2.1 Subjects

Male C57BL/6 mice (initially weighing 20–22 g on arrival) were purchased from the Tianqin Laboratory Animal Technology Co., Ltd., China. A total of 134 mice were used for experiments. Mice were housed in groups of four per cage, maintained on a 12-h light–dark cycle (lights off at 7:00 am, lights on at 7:00 pm) at a room temperature of 20°C–25°C with *ad libitum* access to food and water. All mice were handled 3-min daily for 3 days before behavioral procedures. The current study and its experiments were conducted according to the Guide of Hunan Province for the Care and Use of Laboratory Animals, and experimental protocols were approved by the Local Committee on Animal Care and Use and Protection of the Hunan Normal University (protocol 2021320).

### 2.2 Drugs

The doses of berberine were chosen based on our previous study (Shen et al., 2020). Berberine was resuspended with corn oil to concentrations of 12.5 and 25 mg/ml immediately before use. In the control group, mice were administrated with corn oil (1 ml/kg) intragastrically. Berberine was purchased from J&K Chemical Ltd., Shanghai, China.

### 2.3 Behavioral Procedures

#### 2.3.1 Contextual Fear Conditioning

The CFC procedure was based on our previous study with minor modification (Liu et al., 2015). All mice were handled for 1–2 min per day for 3 days before conditioning. On the training day, they were placed in the conditioning chamber (Shanghai XinRuan Information Technology Co., Ltd.), and three 2-s 0.75-mA foot shocks were delivered on 180, 240, and 300 s, and the mice were allowed to explore the conditioning chamber for an additional 30 s and then removed to their home cages. After removing the mice from the chamber, the chamber was cleaned and scented with 75% alcohol. All training sessions were during the dark cycle (7:00 am–19:00 pm). Freezing behavior was defined as lack of all movement except respiration. The percentage of freezing time was calculated by dividing the freezing time by the total time.

#### 2.3.2 Extinction and Extinction Test

During 5-min daily extinction sessions for 10 days, the conditions were the same as CFC training except that foot shock was no longer delivered. Mice were administrated with berberine (12.5 and 25 mg/kg) or coin oil (1 ml/kg) orally 1 or 6 h after each extinction session.

Extinction tests were performed 2 days after last extinction session; the conditions were the same as the extinction session.

#### 2.3.3 Reinstatement and Spontaneous Recovery

For reinstatement test, a 0.75-mA foot shock was given after the extinction test to reinstate extinguished fear, followed by a 5-min reinstatement test 24 h later in the training chamber.

The spontaneous recovery test was conducted 30 days after the extinction test in the training chamber. The definition of freezing behaviors was the absence of all movement except of respiration.

### 2.4 Statistical Analysis

Data were analyzed using repeated-measures analysis of variance (ANOVA) with appropriate between-subjects factor and within-subjects factor followed by Bonferroni *post hoc* test for each experiment (see Results section). Power analysis was used to calculate appropriately the number of animals at 8) with a statistical power of >85%. The results are presented as the mean ± SEM. *p* < 0.05 was considered statistically significant.

### 2.5 Experimental Design

#### 2.5.1 Experiments 1, 2: The Effect of Berberine Administration After Each Extinction Session on Extinction Training and Subsequent Reinstatement of Recent Fear Memory

**FIGURE 1:**
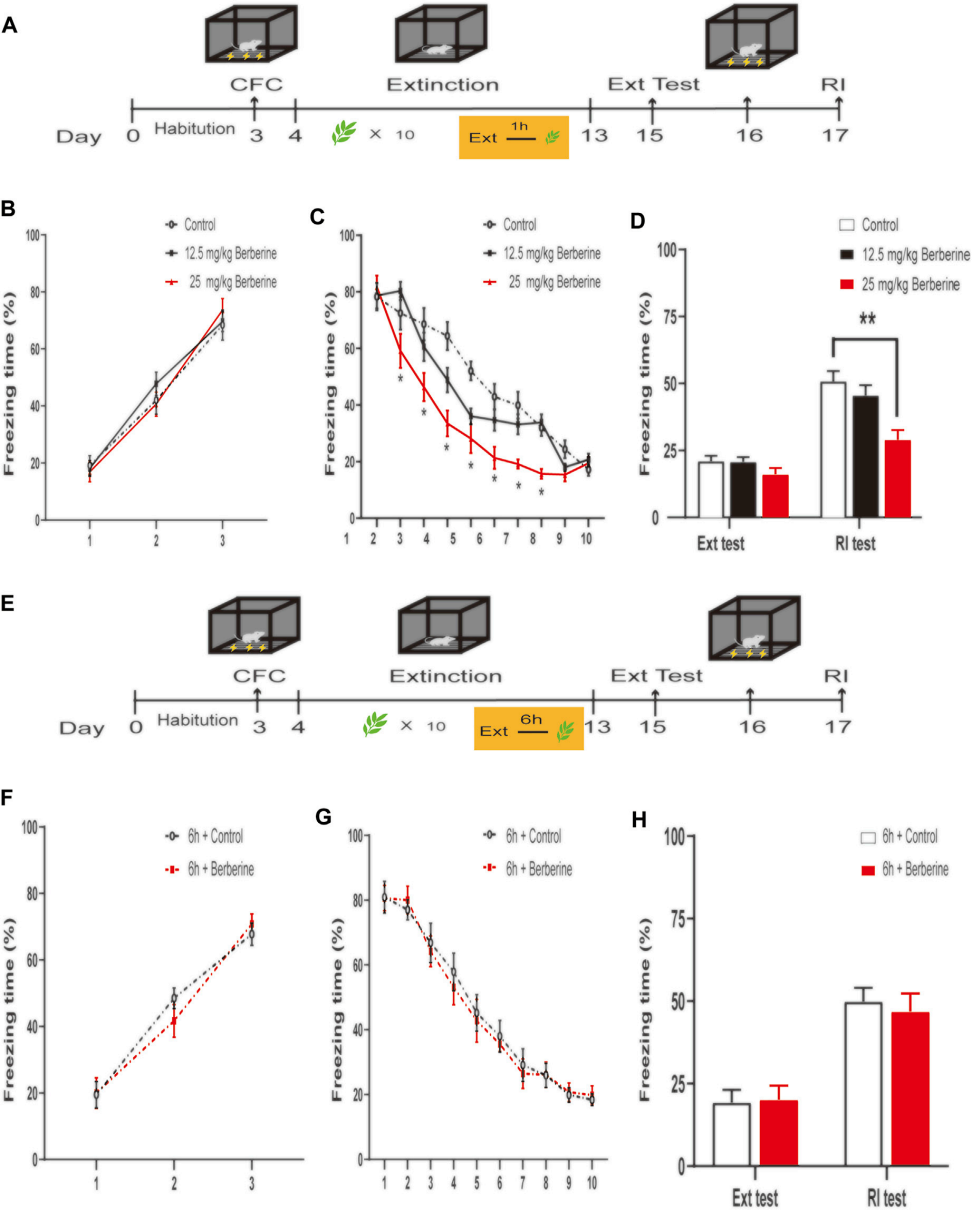
Berberine administration 1 h after extinction enhanced extinction and reduced reinstatement of recent fear. **(A, E)** Timeline of contextual fear conditioning, drug treatment, extinction training, extinction test, and reinstatement test. **(A)** One hour after each extinction training session, corn oil (1 ml/kg, intragastrically [i.g.]) or berberine (12.5 and 25 mg/kg, i.g.) was administered to mice, respectively. **(E)** Six hours after each extinction training session, 1 ml/kg corn oil (i.g.) or 25 mg/kg berberine (i.g.) was administered to mice. **(B, F)** Freezing levels during fear conditioning were similar between different groups. **(C, G)** Freezing behaviors declined during extinction training sessions. **(C)** Repeated-measures ANOVA, effect of the training day, *F*_(9,189)_ = 164.1, *p*0.001; Bonferroni *post hoc* test, *p* < 0.05, during the extinction training days 2–8. **(D, H)** Percentage of freezing time during extinction test (left) and reinstatement test (right). **(D)** Significant freezing time was observed in RI test, *p* < 0.01. *n* = 7–8 mice per group. Data are means ± SEM, *p < 0.05, **p < 0.01, compared with the control group. CFC, contextual fear conditioning; Ext, extinction; RI, reinstatement.

In experiment 1, mice were habituated for 3 days. On day 3, the mice received CFC training, and then were divided into three groups: (1) oral gavage administration of corn oil (1 ml/kg) 1 h after each extinction session (control, *n* = 8); (2) oral gavage administration of berberine (12.5 mg/kg) 1 h after each extinction session (12.5 mg/kg berberine, *n* = 8); (3) oral gavage administration of berberine (25 mg/kg) 1 h after each extinction session (25 mg/kg berberine, *n* = 8). Twenty-four hours after CFC training, the mice underwent 5-min daily extinction training with different treatments for 10 consecutive days (Lacagnina et al., 2019). Two days after the last extinction session, the mice were examined for extinction test. After the extinction test, one single shock was given to reinstate extinguished fear. Twenty-four hours later, mice were conducted for reinstatement test (Figure 1A).

To test whether berberine specifically facilitates consolidation of extinction memory, in experiment 2, mice were divided into two groups after CFC training: (1) oral gavage administration of corn oil (1 ml/kg) 6 h after each extinction session (6 h + control, *n* = 8); (2) oral gavage administration of berberine (25 mg/kg) 6 h after each extinction session (6 h + berberine, *n* = 7). All mice then underwent the same experimental procedures with Figure 1A,E).

#### 2.5.2 Experiments 3, 4: The Effect of Berberine Administration After Each Extinction Session on Extinction Training and Subsequent Spontaneous Recovery of Recent Fear Memory

**FIGURE 2:**
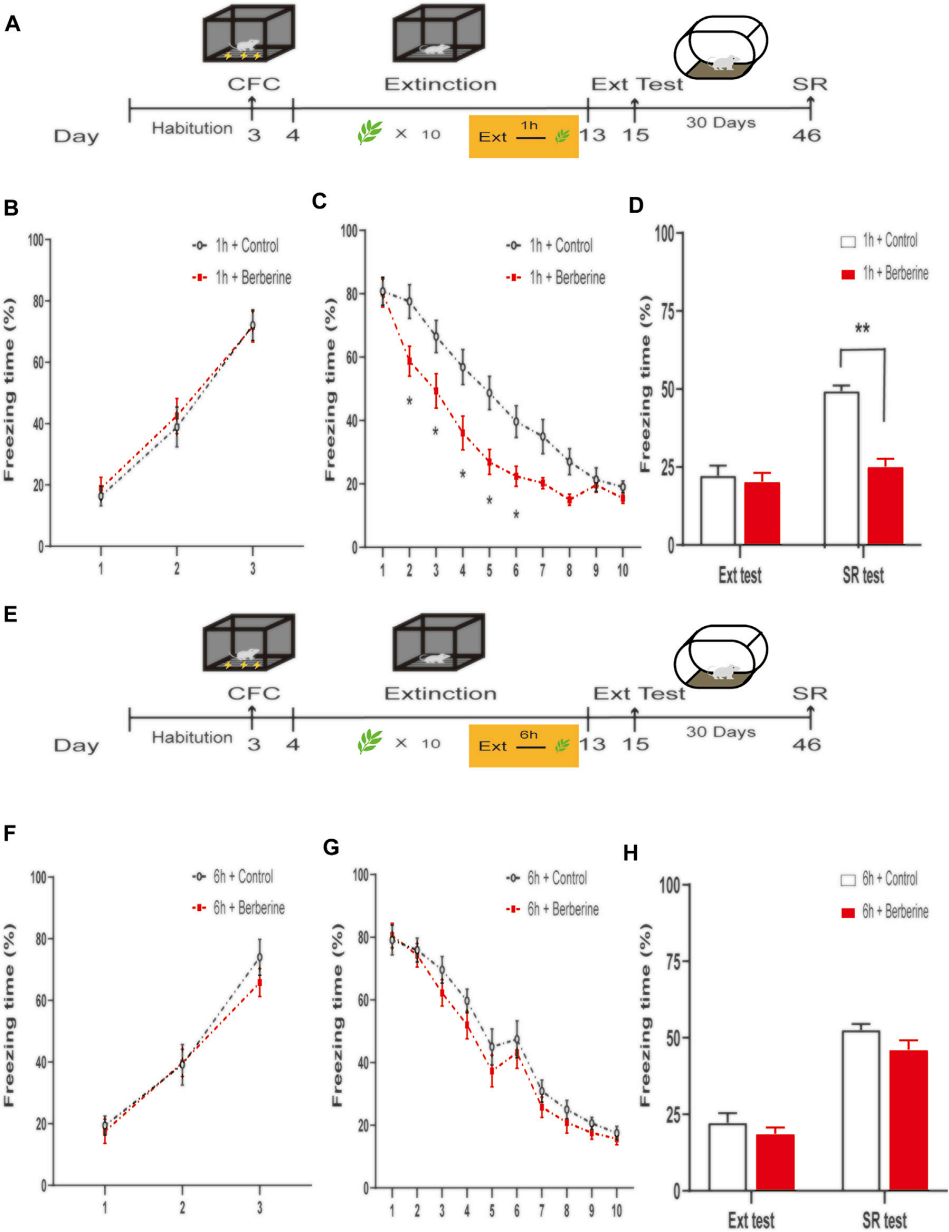
Berberine administration 1 h after extinction enhanced extinction and reduced spontaneous recovery of recent fear. **(A, E)** Timeline of contextual fear conditioning, drug treatment, extinction training, extinction test, and spontaneous recovery test. **(A)** One hour or **(E)** 6 h after each extinction training session, corn oil (1 ml/kg, i.g.) or berberine (25 mg/kg, i.g.) was administered to mice, respectively. **(B, F)** Freezing levels during fear conditioning were similar between different groups. **(C, G)** Freezing behaviors declined during extinction training sessions. **(C)** Repeated-measures ANOVA, effect of the training day, *F*_(9,117)_ = 169.3, *p* < 0.001; Bonferroni *post hoc* test, *p* < 0.05, during the extinction training days 2–6. **(D, H)** Percentage of freezing time during extinction test (left) and spontaneous recovery test (right). **(D)** Significant freezing time was observed in SR test, *p* < 0.01. *n* = 7–8 mice per group. Data are means ± SEM, *p < 0.05, **p < 0.01, compared with 1 h + control group. CFC, contextual fear conditioning; Ext, extinction; SR, spontaneous recovery.

In experiment 3, mice were habituated for 3 days. On day 3, mice received CFC training and then were divided into two groups: (1) oral gavage administration of corn oil (1 ml/kg) 1 h after each extinction session (1 h + control, *n* = 7); (2) oral gavage administration of berberine (25 mg/kg) 1 h after each extinction session (1 h + berberine, *n* = 8). Twenty-four hours after CFC training, mice underwent 5-min daily extinction training with corn oil or berberine administration for 10 consecutive days. Two days after the last extinction session, the mice were examined for extinction test. One month later, the mice were conducted for spontaneous recovery test (Figure 2A).

To examine the effect of delayed administration of berberine on fear extinction and subsequent spontaneous recovery, in experiment 4, mice were divided into two groups after CFC training: (1) oral gavage administration of corn oil (1 ml/kg) 6 h after each extinction session (6 h + control, *n* = 8); (2) oral gavage administration of berberine (25 mg/kg) 6 h after each extinction session (6 h + berberine, *n* = 8). All mice then underwent the same experimental procedures with Figures 2A,E).

#### 2.5.3 Experiments 5, 6: Effect of Berberine Administration After Each Extinction Session on Extinction Training and Subsequent Reinstatement of Remote Fear Memory

**FIGURE 3:**
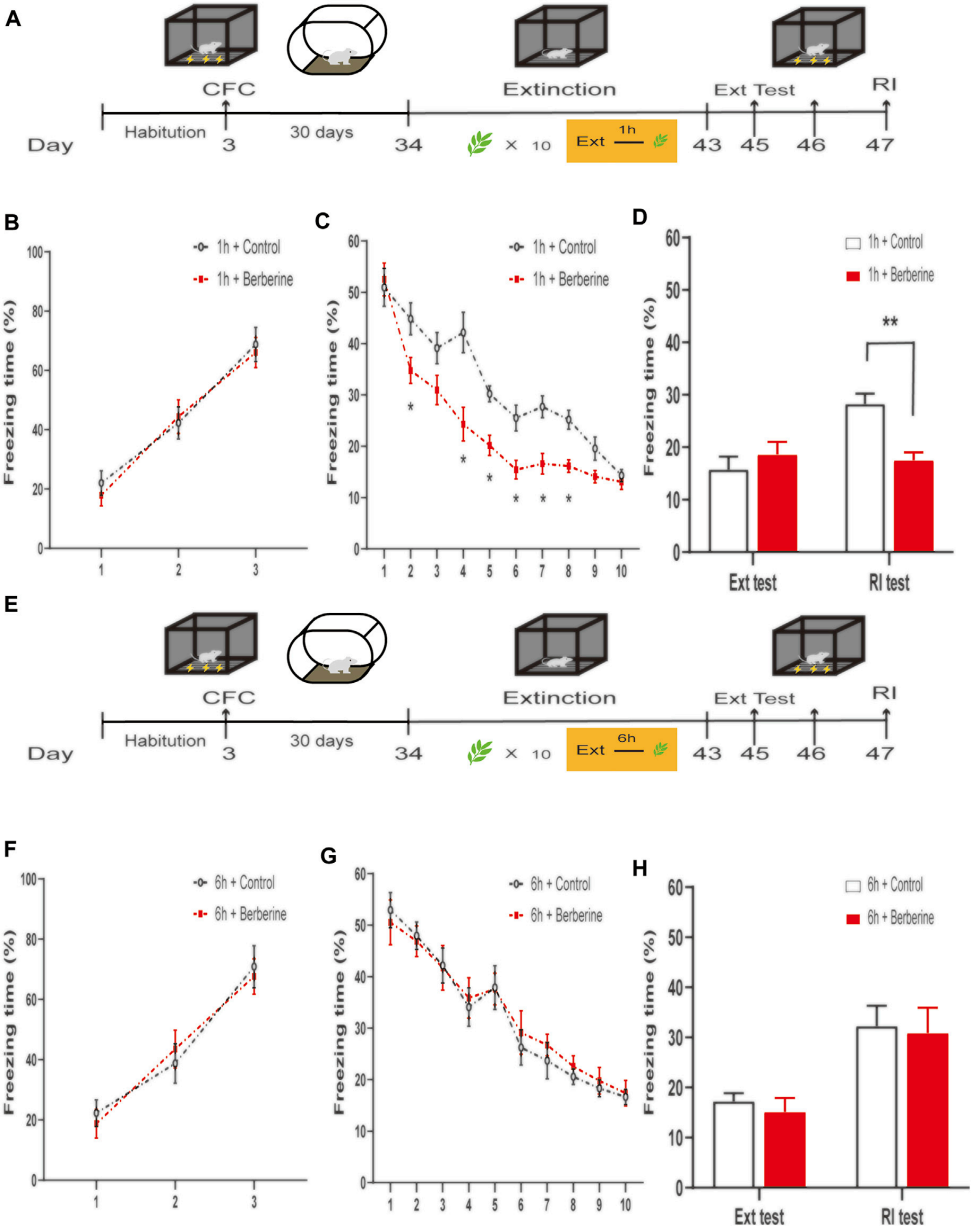
Berberine treatment 1 h after extinction enhanced extinction and reduced reinstatement of remote fear. **(A, E)** Timeline of contextual fear conditioning, drug treatment, extinction training, extinction test, and reinstatement test. **(A)** One hour or **(E)** 6 h after each extinction training session, corn oil (1 ml/kg, i.g.) or berberine (25 mg/kg, i.g.) was administered to mice, respectively. **(B, F)** Freezing levels during fear conditioning were similar between different groups. **(C, G)** Thirty days after CFC, freezing behaviors declined during extinction training sessions. **(C)** Repeated-measures ANOVA, effect of the training day, *F*_(9,126)_ = 233.4, *p* < 0.001; Bonferroni *post hoc* test, *p* < 0.05, during extinction training days 2, 4–8. **(D, H)** Percentage of freezing time during extinction test (left) and reinstatement test (right). **(D)** Significant freezing time was observed in RI test, *p* = 0.0027. *n* = 8 mice per group. Data are means ± SEM, *p < 0.05, **p < 0.01, and compared with 1 h + control group. CFC, contextual fear conditioning; Ext, extinction; RI, reinstatement.

In experiments 5 and 6, the experimental procedure was the same as that in experiments 1 and 2, except that extinction training sessions were performed 30 days rather than 24 h after the CFC training, *n* = 8 in both groups (Figures 3A,E).

#### 2.5.4 Experiments 7, 8: Effect of Berberine Administration After Each Extinction Session on Extinction Training and Subsequent Spontaneous Recovery of Remote Fear Memory

**FIGURE 4:**
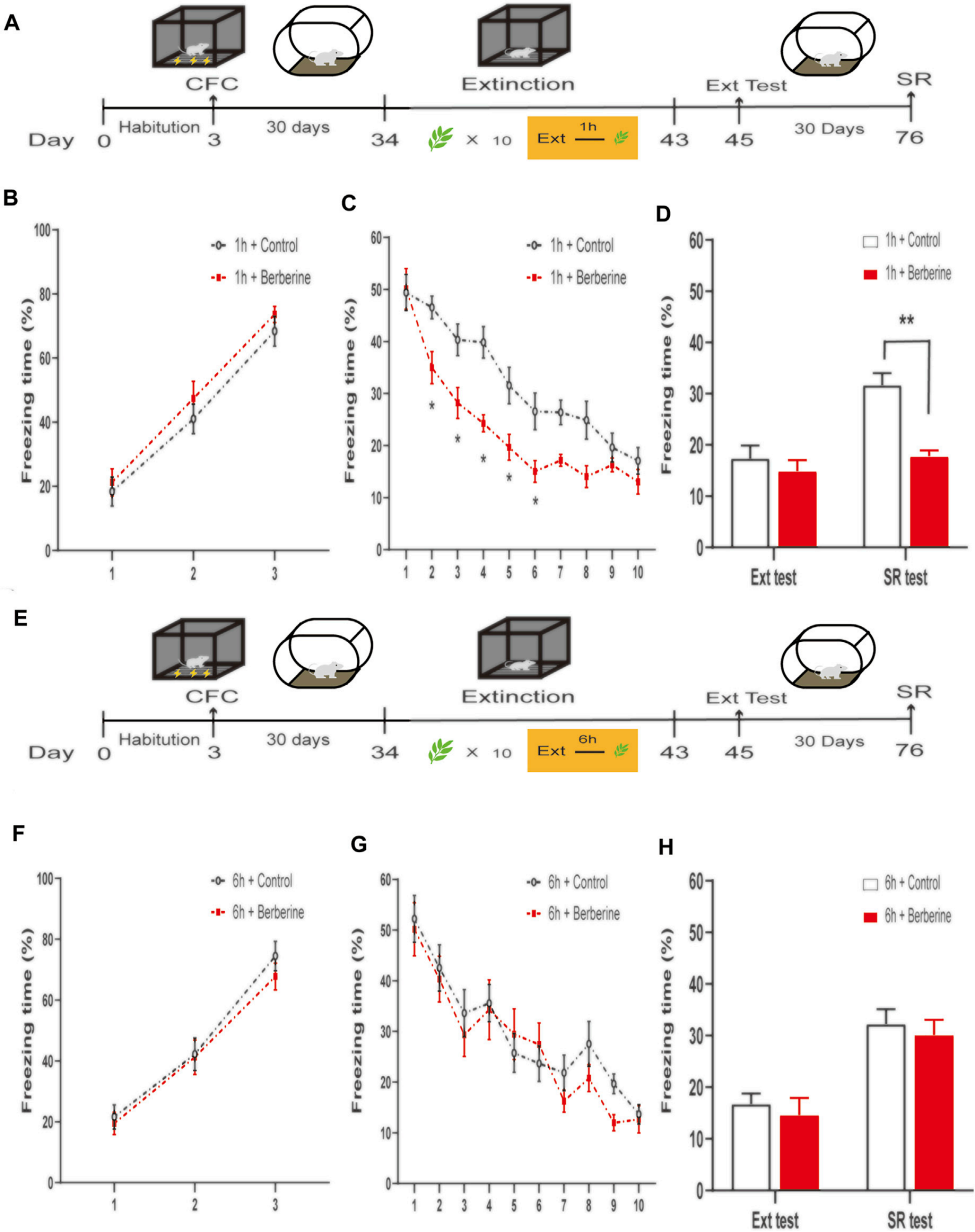
| Berberine treatment 1 h after extinction enhanced extinction and reduced spontaneous recovery of remote fear. **(A, E)** Timeline of contextual fear conditioning, drug treatment, extinction training, extinction test, and spontaneous recovery test. **(A)** One hour or **(E)** 6 h after each extinction training session, corn oil (1 mL/kg, i.g.) or berberine (25 mg/kg, i.g.) was administered to mice, respectively. **(B, F)** Freezing levels during fear conditioning were similar between different groups. **(C, G)** Thirty days after CFC, freezing behaviors declined during extinction training sessions. **(C)** Repeated-measures ANOVA, effect of the training day, *F*_(9,126)_ = 133.8, *p* < 0.001; Bonferroni *post hoc* test, *p* < 0.05, during the extinction training days 2–6. **(D, H)** Percentage of freezing time during extinction test (left) and spontaneous recovery test (right). **(D)** Significant freezing time was observed in SR test, *p* < 0.01. *n* = 8 mice per group. Data are means ± SEM, *p < 0.05, **p < 0.01, and compared with 1 h + control group. CFC, contextual fear conditioning; Ext, extinction; SR, spontaneous recovery.

In experiments 7 and 8, the experimental procedure was the same as that in experiments 3 and 4, except that extinction training sessions were performed 30 days rather than 24 h after the CFC training, *n* = 8 in both groups (Figures 4A,E).

## 3 Results

### 3.1 Berberine Administration 1 h After Extinction Enhanced Extinction and Prevented Subsequent Reinstatement of Recent Fear Memory

In experiment 1, we first investigated the optimal doses and time window of berberine treatment on extinction and subsequent reinstatement of recent fear memory (Figure 1). On the training day, Percent freezing time was significantly different between the first trial and the last trial during fear conditioning (*p* < 0.05; Figure 1B). Extinction training data were analyzed by repeated-measures ANOVA, with the treatment condition (control, 12.5 mg/kg berberine or 25 mg/kg berberine) as a between-subjects factor and different training days as a within-subjects factor. There were significant differences among three groups in the percentage of freezing time during extinction training (main effect of the training day: *F*_(9,189)_ = 164.1, *p* < 0.001; main effect of the treatment condition: *F*_(2,21)_ = 7.328, *p* < 0.01; interaction of training day × treatment condition: *F*_(18,189)_ = 5.167, *p* < 0.001). The Bonferroni *post hoc* test revealed differences between the control and 25 mg/kg berberine groups during the extinction training days 2–8 (*p* < 0.05), suggesting that berberine administration 1 h after extinction promoted extinction of recent fear memory (Figure 1C). There was a significant difference in the percentage of freezing time in reinstatement test between control and 25 mg/kg berberine groups (main effect of the different test day: *F*_(1.21)_ = 275.3, *p* < 0.001; main effect of the different treatment condition: *F*_(2,21)_ = 6.476, *p* < 0.01; interaction of test day× treatment condition: *F*_(2,21)_ = 13.62, *p* < 0.001). *Post hoc* test showed that 25 mg/kg berberine significantly reduced the freezing time compared with the control group in the reinstatement test (*p* < 0.01; Figure 1D). In experiment 2, percent freezing time was significantly different between the first trial and the last trial during fear conditioning (*p* < 0.05; Figure 1F). However, for the freezing time with delayed administration of berberine, there were no significant differences in both extinction training (main effect of the extinction training day: *F*_(9,117)_ = 221.5, *p* < 0.001; main effect of the different treatment condition: *F*_(1,13)_ = 0.029, *p* = 0.8659; interaction of extinction training day × different treatment condition: *F*_(9,117)_ = 0.5734, *p* = 0.8166; Figure 1G) and reinstatement test (main effect of the different test day: *F*_(1.13)_ = 55.38, *p* < 0.001; main effect of the different treatment condition: *F*_(1,13)_ = 0.0467, *p* = 0.8322; interaction of test day × treatment condition: *F*_(1,13)_ = 0.2576, *p* = 0.6203; Figure 1H) between 6 h + control and 6 h + berberine groups.

In summary, these results suggest that berberine administration 1 h after extinction promoted fear extinction and reduced reinstatement of recent fear memory.

### 3.2 Berberine Administration 1 h After Extinction Enhanced Extinction and Prevented Subsequent Spontaneous Recovery of Recent Fear Memory

In experiment 3, we further confirmed the time window of berberine treatment on fear memory extinction and spontaneous recovery of recent fear memory (Figure 2). On the training day, Percent freezing time was significantly different between the first trial and the last trial during fear conditioning (*p* < 0.05; Figure 2B). Extinction training data were analyzed by repeated-measures ANOVA, with the treatment condition (1 h + control or 1 h + berberine) as a between-subjects factor and different training days as a within-subjects factor. There were significant differences between groups in the percentage of freezing time during extinction training (main effect of the training day: *F*_(9,117)_ = 169.3, *p* < 0.001; main effect of the treatment condition: *F*_(1,13)_ = 6.617, *p* < 0.05; interaction of training day × treatment condition: *F*_(9,117)_ = 5.732, *p* < 0.001). The Bonferroni *post hoc* test revealed differences between groups during the extinction training days 2–6 (*p* < 0.05), suggesting that berberine administration 1 h after extinction enhanced extinction of recent fear memory (Figure 2C). There was a significant difference in the percentage of freezing time in spontaneous recovery test between groups (main effect of the different test day: *F*_(1.13)_ = 29.31, *p* < 0.001; main effect of the different treatment condition: *F*_(1,13)_ = 31.7, *p* < 0.01; interaction of test day × treatment condition: *F*_(1,13)_ = 14.14, *p* < 0.01). *Post hoc* test showed that berberine administration 1 h after extinction significantly reduced the freezing time compared with the 1 h + control group in the spontaneous recovery test (*p* < 0.01; Figure 2D). In experiment 4, percent freezing time was significantly different between the first trial and the last trial during fear conditioning (*p* < 0.05; Figure 2F). However, for freezing time with delayed administration of berberine, there were no significant differences in both extinction training (main effect of the extinction training day: *F*_(9,126)_ = 217.1, *p* < 0.001; main effect of the different treatment condition: *F*_(1,14)_ = 0.7743, *p* = 0.3937; interaction of extinction training day × different treatment condition: *F*_(9,126)_ = 0.8957, *p* = 0.5313; Figure 2G) and spontaneous recovery test (main effect of the different test day: *F*_(1.14)_ = 101.6, *p* < 0.001; main effect of the different treatment condition: *F*_(1,14)_ = 4.4883, *p* = 0.0443; interaction of test day × treatment condition: *F*_(1,14)_ = 0.2508, *p* = 0.6243; Figure 2H) between 6 h + control and 6 h + berberine groups.

These results indicate that berberine administration 1 h after extinction enhanced extinction rates and reduced spontaneous recovery of recent fear memory. Experiments 1, 2, 3, and 4 data verify the hypothesis that berberine combined with extinction training enhance extinction memory and prevent the return of fear.

### 3.3 Berberine Treatment 1 h After Extinction Enhanced Extinction and Prevented Subsequent Reinstatement of Remote Fear Memory

In experiment 5, we examined whether the facilitating effect of berberine on extinction was also applied to remote fear memory. One month after the CFC training, the mice were administered control or berberine 1 h after each extinction session and then tested for extinction and reinstatement of extinguished fear (Figure 3). On the training day, Percent freezing time was significantly different between the first trial and the last trial during fear conditioning (*p* < 0.05; Figure 3B). Extinction training data were analyzed by repeated-measures ANOVA, with the treatment condition as a between-subjects factor and different training days as a within-subjects factor. There was significant difference between 1 h + control and 1 h + berberine groups in the percentage of freezing time during extinction training (main effect of the training day: *F*_(9,126)_ = 233.4, *p* < 0.001; main effect of the treatment condition: *F*_(1,14)_ = 6.814, *p* < 0.05; interaction of training day × treatment condition: *F*_(9,126)_ = 12.23, *p* < 0.001). The Bonferroni *post hoc* test revealed differences between 1 h + control and 1 h + berberine groups during extinction training days 2, 4–8 (*p* < 0.05), suggesting that berberine administration 1 h after extinction enhanced extinction of remote fear memory (Figure 3C). Also, there was a significant difference in the percentage of freezing time in reinstatement test between 1 h + control and 1 h + berberine groups (main effect of the different test day: *F*_(1.14)_ = 5.842, *p* < 0.05; main effect of the different treatment condition: *F*_(1,14)_ = 4.369, *p* = 0.05; interaction of test day × treatment condition: *F*_(1,14)_ = 8.382, *p* < 0.05). *Post hoc* test showed that berberine administration 1 h after extinction significantly reduced the freezing time compared with the 1 h + control group in the reinstatement test (*p* < 0.01; Figure 3D). In experiment 6, percent freezing time was significantly different between the first trial and the last trial during fear conditioning (*p* < 0.05; Figure 3F). However, for freezing time with delayed administration of berberine, there were no significant differences in both extinction training (main effect of the extinction training day: *F*_(9,126)_ = 102.9, *p* < 0.001; main effect of the different treatment condition: *F*_(1,14)_ = 0.0389, *p* = 0.8469; interaction of extinction training day × different treatment condition: *F*_(9,126)_ = 0.5504, *p* = 0.5504; Figure 3G) and reinstatement test (main effect of the different test day: *F*_(1.14)_ = 17.49, *p* < 0.001; main effect of the different treatment condition: *F*_(1,14)_ = 0.2414, *p* = 0.6308; interaction of test day × treatment condition: *F*_(1,14)_ = 0.01, *p* = 0.9202; Figure 3H) between 6 h + control and 6 h + berberine groups.

These results indicate that berberine administration 1 h after extinction enhanced extinction learning and prevented reinstatement of remote fear memory.

### 3.4 Berberine Treatment 1 h After Extinction Enhanced Extinction and Prevented Subsequent Spontaneous Recovery of Remote Fear Memory

In experiment 7, we further investigated the effects of berberine treatment on extinction and spontaneous recovery of remote fear memory (Figure 4). On the training day, percent freezing time was significantly different between the first trial and the last trial during fear conditioning (*p* < 0.05; Figure 4B). Extinction training data were analyzed by repeated-measures ANOVA, with the treatment condition (1 h + control or 1 h + berberine) as a between-subjects factor and different training days as a within-subjects factor. There was significant difference between groups in the percentage of freezing time during extinction training (main effect of the training day: *F*_(9,126)_ = 133.8, *p* < 0.001; main effect of the treatment condition: *F*_(1,14)_ = 6.711, *p* = 0.0214; interaction of training day × treatment condition: *F*_(9,126)_ = 6.762, *p* < 0.001). The Bonferroni *post hoc* test revealed differences between groups during the extinction training days 2–6 (*p* < 0.05), suggesting that berberine administration 1 h after extinction enhanced extinction of remote fear memory (Figure 4C). There was a significant difference in the percentage of freezing time in spontaneous recovery test between groups (main effect of the different test day: *F*_(1.14)_ = 31.76, *p* < 0.001; main effect of the different treatment condition: *F*_(1,14)_ = 10.29, *p* < 0.01; interaction of test day × treatment condition: *F*_(1,14)_ = 14.05, *p* < 0.01). *Post hoc* test showed that berberine administration 1 h after extinction significantly reduced the freezing time compared with the 1 h + control group in the spontaneous recovery test (*p* < 0.01; Figure 4D). In experiment 8, percent freezing time was significantly different between the first trial and the last trial during fear conditioning (*p* < 0.05; Figure 4F). However, for freezing time with delayed administration of berberine, there were no significant differences in both extinction training (main effect of the extinction training day: *F*_(9,126)_ = 38.39, *p* < 0.001; main effect of the different treatment condition: *F*_(1,14)_ = 0.3073, *p* = 0.5881; interaction of extinction training day × different treatment condition: *F*_(9,126)_ = 1.069, *p* = 0.3899; Figure 4G) and spontaneous recovery test (main effect of the different test day: *F*_(1.14)_ = 43.97, *p* < 0.001; main effect of the different treatment condition: *F*_(1,14)_ = 0.4316, *p* = 0.5218; interaction of test day × treatment condition: *F*_(1,14)_ < 0.01, *p* > 0.99; Figure 4H) between 6 h + control and 6 h + berberine groups.

These findings indicate that berberine administration 1 h after extinction enhanced extinction rates and reduced spontaneous recovery of remote fear memory. All experiments data support the hypothesis that berberine-induced enhancement of extinction memory persistently attenuate fear memory in both recent and remote fear memory.

## Discussion

This study provides evidence for the facilitating effects of berberine on fear extinction using the classical CFC paradigm. The main findings of this study are as follows: (1) oral administration of berberine 1 h after each extinction session enhanced extinction rates and reduced the reinstatement of freezing behavior in both recent and remote fear memories; (2) the positive effects of berberine enhancing the extinction memory persisted for at least 30 days; (3) delayed administration of berberine 6 h after each extinction session had no effects on extinction training, reinstatement, and spontaneous recovery of fear-associated behaviors. Altogether, these results indicate that berberine treatment combined with extinction training during the critical time window of extinction memory consolidation leads to persistent attenuation of fear memories and provide evidence for the development of berberine-based postextinction pharmacological interventions for PTSD.

The modulation of pharmacological or behavioral interventions on extinction may have two consequences: extinction or the original fear memory traces (Richardson et al., 2004; Quirk et al., 2010; Kida, 2019; Bouton et al., 2021). We found that extinguished fear can reemerge in reinstatement and spontaneous recovery test, indicating that the berberine-induced enhancement of extinction did not erase but compete the original fear memory. The formation of extinction memory resulted in suppression of original memory, and extinction memory also involved acquisition, consolidation, and storage processes (Millan et al., 2011; Orsini and Maren, 2012; Maren et al., 2013; Careaga et al., 2016; Knox, 2016). After acquisition, the memory undergoes a consolidation process that typically lasts for 6 h to be completely stored (Bourtchouladze et al., 1998; Schafe et al., 1999; Mcgaugh, 2000; Dudai, 2004; Lalumiere et al., 2017). Berberine treatment 1 h but not 6 h after extinction enhanced extinction memory, indicating that berberine promoted the consolidation of extinction memory. Moreover, berberine treatment 1 h but not 6 h after extinction inhibited subsequent tests of fear freezing behavior, demonstrating that berberine enhanced the consolidation of extinction memory rather than influenced the storage of extinction memory trace. Many studies have shown that cognitive enhancers administered before or after extinction facilitated extinction learning (Walker et al., 2002; Parnas et al., 2005; Lee et al., 2006; Makkar et al., 2010). Nevertheless, most of them do not apply to the clinical population because of their health-related side effects (Steckler and Risbrough, 2012; Singewald et al., 2015). Berberine, an OTC medication in China, has been verified well for safety in clinical trials (Gupte, 1975). Besides, previous studies have shown that berberine affected the DA and *N*-methyl-d-aspartate (NMDA) systems, which is the molecular mechanism of cognitive enhancers (Yoo et al., 2006; Kaplan and Moore, 2011; Kawano et al., 2015; Lee et al., 2018). Shen et al. (2020) demonstrated that extinction training combined with berberine treatment facilitated the extinction of drug memory. However, at least to our knowledge, the effect of berberine treatment on fear memory has not been reported.

In this study, we found that berberine facilitated extinction of fear-related behaviors, as well as reduced reinstatement, and spontaneous recovery of extinguished fear. But what might account for this phenomenon? For the accelerated extinction rates, we speculate that berberine acts as a cognitive enhancer to facilitate the consolidation of fear extinction memory. Growing evidence supports the modulatory effects of berberine on NMDA and DA systems (Kulkarni and Dhir, 2008; Vahdati Hassani et al., 2016). In fact, it has been widely accepted that NMDA and DA receptor agonists facilitated extinction of conditioned fear (Quirk et al., 2006; Peters et al., 2009; Davis, 2011), which provides an underlying pharmacological mechanism of the berberine’s enhancement effect in our findings. Previous studies showed that berberine is an antagonist of both DA D1- and D2-like receptors (Kawano et al., 2015); however, only the D2 receptor antagonist has such a reinforcement effect of extinction but the opposite function of D1 (Ponnusamy et al., 2005). This led to the question of the exact receptor that may be affected by berberine, and further study should focus on the relevance between D1 and D2 receptors to elucidate the potential mechanisms underlying the berberine-induced fear extinction enhancement. For the suppression of reinstatement and spontaneous recovery of fear, the most likely explanation is that berberine-induced enhancement of extinction memory takes advantage in competing with the original fear memory. Extinction memory learning can compete with the original fear memory, which results in transitory but not persistent suppression of fear-related behaviors (Furini et al., 2014; Izquierdo et al., 2016). Surprisingly, berberine treatment combined with extinction training significantly suppressed the remote fear memory, indicating that berberine not only enhanced the extinction rates but also possibly gradually altered the plasticity-related proteins to produce such a long-term effect. Shen et al. also observed the alterations of plasticity-related proteins with berberine treatment, and future studies are needed to identify specific proteins to investigate the underlying molecular mechanisms of persistent suppression in remote fear of postextinction berberine treatment.

Notably, in the present data, the difference during extinction between the control and berberine groups that received the substance 1 h after extinction training disappeared in the last two to four trials in all experiments. Two potential explanations account for the interesting phenomenon. One is that berberine produces a transient effect of the substance during extinction training, and the other is the floor effect; the freezing behavior of the mice in the berberine groups has reached the standards of extinction in the last two to four trials so that it blurs the difference between groups. We prefer the second explanation but not the transient effect because such manipulation persistently suppresses the reinstatement and spontaneous recovery of original fear memories.

Although we demonstrated that berberine promoted extinction of fear-related behaviors and persistently attenuated fear memory by enhancing the extinction memory, further research is needed to explore the potential direct targets of berberine’s effects. More recently, there is renewed attention to berberine because of its beneficial effects on various neurodegenerative and neuropsychiatric disorders, including anticonvulsant, antidepressant, and anti-Parkinson (Bhutada et al., 2010; Fan et al., 2017; Wang et al., 2021). All these effects of berberine may be attributed to its ability to regulate several neurotransmitter systems, like NMDA and serotonin (Kong et al., 2001; Castillo et al., 2005; Yoo et al., 2006). Moreover, NMDA receptors are important in learning and memory and in experience-dependent forms of plasticity such as LTP, and play a role in the extinction of the fear startle response and CFC (Ledgerwood et al., 2003; Davis et al., 2006). Also, a study examined serotonin reuptake genes in fear conditioning and extinction paradigms in mice, and they found that fear conditioning and extinction were normal but a deficit in extinction recall, indicating that serotonin reuptake may be a mechanism for fear extinction (Wellman et al., 2007). Therefore, we speculate that the suppression of berberine on fear-related behaviors may be related to NMDA and serotonin receptors. Undeniably, we first found the potential therapeutic potential of berberine in fear-related disorders, but more research is needed to reveal the underlying neurochemical and molecular mechanisms of the beneficial effects of postextinction berberine treatment.

Exposure therapy greatly impacts the prognosis of patients with anxiety-related disorders (Singewald et al., 2015; Foa and Mclean, 2016). In our study, considering that berberine promoted extinction of fear-related behaviors, it indicates that berberine may be potentially therapeutic in all anxiety-related disorders based on exposure therapy. Further studies should identify berberine’s enhancement of extinction in pathological fear and anxiety psychiatric conditions, such as social phobia, agoraphobia, obsessive–compulsive disorder, and generalized anxiety (World Health Organization, 2009; Edition, 2013), to investigate whether berberine has such extinction enhancement effect in various types of anxiety-related disorders.

In conclusion, we found that berberine administration orally 1 h after extinction facilitated extinction and led to persistent attenuation of both recent and remote fear memories. Postextinction berberine treatment might be a potential and promising pharmacological intervention for anxiety-related disorders. Further research is needed to elucidate the mechanisms underlying the beneficial effects of berberine on fear-related behaviors, and it is necessary to determine whether the therapeutic effects of berberine are applicable to the clinical population.

## Data Availability

The original contributions presented in the study are included in the article/Supplementary Material, further inquiries can be directed to the corresponding authors.
